# Establishment of CRISPR/Cas9 Genome Editing in Witloof (*Cichorium intybus* var. *foliosum*)

**DOI:** 10.3389/fgeed.2020.604876

**Published:** 2020-12-21

**Authors:** Charlotte De Bruyn, Tom Ruttink, Tom Eeckhaut, Thomas Jacobs, Ellen De Keyser, Alain Goossens, Katrijn Van Laere

**Affiliations:** ^1^Plant Sciences Unit, Flanders Research Institute for Agriculture, Fisheries and Food (ILVO), Melle, Belgium; ^2^Department of Plant Biotechnology and Bioinformatics, Ghent University, Ghent, Belgium; ^3^Center for Plant Systems Biology, Flanders Institute for Biotechnology (VIB), Ghent, Belgium

**Keywords:** gene editing, protoplast transfection, *Cichorium intybus*, sesquiterpene lactones, phytoene desaturase, HiPlex amplicon sequencing

## Abstract

*Cichorium intybus* var. *foliosum* (witloof) is an economically important crop with a high nutritional value thanks to many specialized metabolites, such as polyphenols and terpenoids. However, witloof plants are rich in sesquiterpene lactones (SL) which are important for plant defense but also impart a bitter taste, thus limiting industrial applications. Inactivating specific genes in the SL biosynthesis pathway could lead to changes in the SL metabolite content and result in altered bitterness. In this study, a CRISPR/Cas9 genome editing workflow was implemented for witloof, starting with polyethylene glycol (PEG) mediated protoplast transfection for CRISPR/Cas9 vector delivery, followed by whole plant regeneration and mutation analysis. Protoplast transfection efficiencies ranged from 20 to 26 %. A CRISPR/Cas9 vector targeting the first exon of the *phytoene desaturase* (*CiPDS*) gene was transfected into witloof protoplasts and resulted in the knockout of *CiPDS*, giving rise to an albino phenotype in 23% of the regenerated plants. Further implementing our protocol, the SL biosynthesis pathway genes *germacrene A synthase (GAS), germacrene A oxidase (GAO)*, and *costunolide synthase (COS)* were targeted in independent experiments. Highly multiplex (HiPlex) amplicon sequencing of the genomic target loci revealed plant mutation frequencies of 27.3, 42.7, and 98.3% in regenerated plants transfected with a CRISPR/Cas9 vector targeting *CiGAS, CiGAO*, and *CiCOS*, respectively. We observed different mutation spectra across the loci, ranging from consistently the same +1 nucleotide insertion in *CiCOS* across independent mutated lines, to a complex set of 20 mutation types in *CiGAO* across independent mutated lines. These results demonstrate a straightforward workflow for genome editing based on transfection and regeneration of witloof protoplasts and subsequent HiPlex amplicon sequencing. Our CRISPR/Cas9 workflow can enable gene functional research and faster incorporation of novel traits in elite witloof lines in the future, thus facilitating the development of novel industrial applications for witloof.

## Introduction

Witloof belongs to the species *Cichorium intybus*, a member of the Asteraceae family. Within the *Cichorium* genus, three groups are distinguished: root chicory, leaf chicory and Witloof (Raulier et al., 2015). Root chicory, also known as industrial chicory (*C. intybus* var. *sativum*), is characterized by a large tap root and is mainly grown for inulin production. The leaf chicory group can be divided into three subgroups: Sugarloaf (*C. intybus* var. *porphyreum*), Radicchio (*C. intybus* var. *latifolium*), and Catalogne (*C. intybus* var. *intybus* or *C. intybus* var. *sylvestre*); they consist of leafy vegetables that can be consumed fresh or cooked. Witloof, also referred to as Witlof, Belgian endive, Chicon, or *C. intybus* var. *foliosum*, is a biennial plant that, without forcing conditions, produces a taproot and a rosette of leaves in the first year of growth and, following a period of cold exposure, develops a floral meristem in the second year of growth. By cultivating the tap root under artificial conditions, a vegetable composed of tightly packed white leaves is produced, known as witloof. It is a traditional Belgian crop rich in nutritionally relevant compounds, such as polyphenols and terpenoids, which have positive effects on human health because of the biological and pharmacological activities of their specialized metabolites (Street et al., 2013). Consequently, it is a crop with an important economic value, which has led to large scale cultivation (2.100 ha annually in Belgium). However, the bitter taste limits the use of witloof as it has a negative influence on consumer acceptability (Drewnowski and Gomez-Carneros, 2000). Modifying the bitterness in witloof can lead to product differentiation by creating a more diverse range of flavors enabling consumers to choose between less or more bitter cultivars, maximizing the acceptance and economic impact and creating new market opportunities.

Sesquiterpene lactones (SLs) are the specialized metabolites responsible for the bitter taste and play an important role in plant defense against herbivores and pathogens (Peters et al., 1997). Within the SLs, different classes are recognized, with the guaianolides being the most important with regard to bitterness (de Kraker et al., 1998). This class comprises lactucin, deoxylactucin, lactucopicrin, and their derivatives (Chadwick et al., 2013). They all originate from the cytosolic mevalonate pathway that leads to the production of the building blocks isopentenyl pyrophosphate (IPP) and dimethylallyl pyrophosphate (DMAPP). When DMAPP reacts with two units of IPP, farnesyl diphosphate (FDP) is formed, which is further converted to costunolide via the enzymes germacrene A synthase (GAS), germacrene A oxidase (GAO), and costunolide synthase (COS) (de Kraker et al., 1998; Liu et al., 2011). The genes encoding GAS, GAO and COS have previously been cloned from chicory (Bouwmeester, 2002; Liu et al., 2011) and are of significant importance to control the biosynthesis of SLs and hence the bitterness of witloof.

In traditional witloof breeding, the stable integration of specific traits in elite lines takes a minimum of 10 years, making breeding programs to improve yield and nutritional properties time-consuming and labor intensive. CRISPR/Cas9 [clustered regularly interspaced short palindromic repeat (CRISPR) associated nuclease (CAS)] genome editing, which has recently been applied on a number of plant species (Manghwar et al., 2019; Zhang et al., 2019), can be a valuable tool to study the function of genes involved in specific specialized metabolite biosynthetic pathways, such as the SL pathway, and thus identify the genomic target loci underlying crop improvement. Moreover, CRISPR/Cas9 genome editing may offer more straightforward breeding opportunities to generate new varieties within a shorter time period by targeted introduction of functional sequence diversity in elite lines (Zhang et al., 2019). The CRISPR/Cas9 system enables the alteration of specific DNA sequences to achieve gene modifications. The Cas9 endonuclease uses a guide RNA (gRNA) with a 20 nucleotide spacer sequence to recognize a complementary target DNA site (the protospacer) upstream of a protospacer adjacent motif (PAM) in the genomic DNA. Upon recognition, Cas9 generates a double-stranded DNA break (DSB) between the 3rd and 4th nucleotides on the 5′ side of the PAM (Jinek et al., 2012). These DSBs are typically repaired through non-homologous end joining (NHEJ), whereby imperfect repair results in small insertions/deletions (indels) and/or substitutions (SNPs) at the target region (Manghwar et al., 2019). Such mutations in protein coding sequences may result in premature stop codons downstream of the indel, inducing the elimination of the protein product. Changes in the regulation of gene expression or the activity of the encoded protein are also possible.

The CRISPR/Cas9 system is often delivered through a vector into plant cells. Hereby, two main delivery methods are commonly used: *Agrobacterium*-mediated transformation and protoplast transfection. Using *Agrobacterium* transformation, the CRISPR/Cas9 system is typically stably integrated into the plant genome, whereas protoplast transfection allows for transient expression of the CRISPR/Cas9 system (Zhang et al., 2019). As protoplasts are cells without a cell wall, DNA uptake can readily occur through the plasma membrane using polyethylene glycol (PEG) (Zhang et al., 2019). As a result, a high quantity (>50,000) of cells can be simultaneously transfected and plants can be regenerated from single cells (Jaganathan et al., 2018). Furthermore, the chance of stable integration of vector DNA into the genome is reduced and off-target effects are decreased (Zhang et al., 2016). Working with protoplasts also allows the implementation of a DNA-free transfection method using pre-assembled ribonucleoprotein complexes (RNPs) instead of vector DNA (Wook Woo et al., 2015). Because the delivery vectors are not stably integrated into the plant genome, using a transient transfection method does not allow the use of typical plant selection markers (e.g., kanamycin, hygromycin, *bar* gene). Hence, high protoplast transfection and mutation efficiencies are required. Isolating and, especially, regenerating protoplasts can be very challenging, thus hampering the strategy in many genera and species. However, a successful protoplast isolation and regeneration method has previously been developed for *Cichorium* (Deryckere et al., 2012). Recently, protoplasts of *C. intybus* have been successfully transfected and mutated in the *phytoene desaturase* (*CiPDS*) gene, using a CRISPR/Cas9 PEG-mediated transfection protocol, resulting in 4.5% of the regenerated plants with an albino phenotype (Bernard et al., 2019).

In this study, we report an efficient CRISPR/Cas9 genome editing workflow for witloof based on PEG-mediated protoplast transfection, transient expression of CRISPR/Cas9 vectors and whole plant regeneration. We first developed a protoplast transfection and regeneration protocol using a CRISPR/Cas9 vector to induce mutations in the *CiPDS* gene, leading to the regeneration of several independent albino plantlets. To further implement the genome editing technique, we used our CRISPR/Cas9 protocol to induce mutations in *CiGAS, CiGAO*, and *CiCOS*, three genes known to be involved in the SL biosynthesis pathway. Highly multiplex (HiPlex) amplicon sequencing was used to analyze the genomic target loci in the regenerated plants and revealed a variety of mutated alleles and targeted knockouts, indicating the potential of our CRISPR/Cas9 workflow.

## Materials and Methods

### Plant Material

Plant materials of witloof *C. intybus* var. *foliosum* “Van Hamme” and “Topmodel” were provided by COSUCRA (Belgium). Roots of *in vivo* plants of the selected *Cichorium* varieties were rinsed with water, grated on the outside and cut into slices of 1 cm. The slices were rinsed for 1 min in 70% ethanol, sterilized in 2.5% NaOCl, and rinsed in sterile water. Next, the slices were cut into pieces of 1–2 cm^3^ and transferred to solid plant medium [4.4 g.L^−1^ Murashige and Skoog medium (Murashige and Skoog, 1962), 45 g.L^−1^ sucrose, 8 g.L^−1^ plant tissue culture agar No. 4 (Neogen, Lansing, Michigan, United States), pH 6] at 23 ± 2°C under a 16/8 h (light/dark) photoperiod at 40 μmol.m^−2^.s^−1^ photosynthetic active radiation. After shoot induction, plants were transferred to new solid plant medium (4.4 g.L^−1^ Murashige and Skoog medium + vitamins, 20 g.L^−1^ sucrose, 7 g.L^−1^ plant tissue culture agar No. 4, pH 6) and subcultured every 6 weeks.

### CRISPR/Cas9 Vector Construction

Guide RNAs for *CiPDS* (MK455771), *CiGAS* (AF498000.1)*, CiGAO* (ADF43080), and *CiCOS* (G3GBK0) were designed in the first half of the CDS using Geneious 10.2.6 (http://www.geneious.com) and were selected based on high on-target activity scores (Doench et al., 2014).

An overview of de vector constructions can be found in Supplementary Figure 1. A first step in the construction of a *Cas9* destination vector, was to “domesticate” a common high-copy entry vector by removing a *Bbs*I restriction site in the backbone. Hence, digestion of pEN-L1-AG-L2 (Houbaert et al., 2018) with *Apa*I and *Bbs*I was followed by ligation with a double-stranded linker (Supplementary Table 1) with a mutated *Bbs*I site, generating the vector pEN-L1-AG-L2,\ (-Bbs1). In a second step, six entry clones pGGA004, pGGB003, pGGD002, pGGE001 (Lampropoulos et al., 2013), pGG-C-Cas9PTA^*^-D, pGG-F-AtU6-26-BbsI-BbsI-G (Decaestecker et al., 2019), and annealed *Bsa*I oligos 9 and 10 (Supplementary Table 1) were assembled into pEN-L1-AG-L2,\(-Bbs1), generating the Cas9 destination vector pCDB-Cas9. Prior to assembly, pGG-F-AtU6-26-BbsI-BbsI-G was digested with *Bbs*I. The Golden Gate reaction was performed as previously described (Decaestecker et al., 2019). The pCDB-Cas9 destination vector contains two *Bsa*I restriction sites between the AtU6-26 promotor and the gRNA scaffold to enable one-step Golden Gate assembly of new gRNAs. Next, the *ccdB* gene and chloramphenicol resistance marker (CmR) (Decaestecker et al., 2019) was added between the AtU6-26 and scaffold elements to further streamline the cloning of new gRNAs. pCDB-Cas9 was digested with *Bsa*I, after which the ccdB/CmR insert was ligated to obtain the unarmed gRNA destination vector pCDB-Cas9-ccdB.

A similar destination vector, pCDB-Cas9-GFP-ccdB was generated containing a Green Fluorescent Protein–Nuclear Localization Signal (*GFP-NLS*)-tag translationally fused to the *Cas9* C-terminus. The entry vectors pGGA004, pGGB003, pGGD001, pGGE001 (Lampropoulos et al., 2013), pGG-C-Cas9PTA-D, pGG-F-AtU6-26-AarI-AarI-G (Decaestecker et al., 2019), were combined into the vector pEN-L1-AG-L2,\(-Bbs1) to construct the vector pCDB-Cas9-GFP. The vector was digested with *Aar*I and the ccdB/CmR insert was added to obtain the unarmed gRNA destination vector pCDB-Cas9-GFP-ccdB.

Oligos 1−8 (Supplementary Table 1) containing the gRNA sequences and 5′ overlap sequences (5′-ATTG-N_20_-3′ and 5′-AAAC-N_20_(reverse complement)-3′) were annealed and cloned into the unarmed gRNA destination vectors pCDB-Cas9-cddB and pCDB-Cas9-GFP-ccdB, as previously described (Decaestecker et al., 2019). This yielded the vectors pCDB-Cas9-PDS, pCDB-Cas9-GFP-PDS, pCDB-Cas9-GAS, pCDB-Cas9-GAO, and pCDB-Cas9-COS (Supplementary Table 2). All vectors were verified using colony PCR with primer20 and primer24 (Supplementary Table 1), followed by a *Hinc*II restriction digest. Vector DNA was extracted using QIAGEN Plasmid Maxi Kit and the vector gRNA sites were analyzed using Sanger sequencing with primer20. All vectors and their size are listed in Supplementary Table 2 and are available via the Gateway vector website https://gatewayvectors.vib.be.

### Protoplast Isolation, Transfection, and Regeneration

#### Protoplast Isolation and Transfection

Witloof protoplasts were isolated from young and healthy leaves from *in vitro* maintained plants as previously described (Deryckere et al., 2012). Protoplast suspensions were diluted to 500,000 protoplasts.mL^−1^ and 100 μL was added to a minimum of 10 μg of vector. Next, 120 μL PEG3350 solution [400 g.L^−1^ PEG3350, 72.8 g.L^−1^ mannitol, 23.6 g.L^−1^ Ca(NO_3_)_2_.4H_2_O, pH 6] was added to the solution, gently mixed and samples were incubated in the dark for 10 min at room temperature. The transfection reaction was stopped by adding 600 μL of W5 medium (8.77 g.L^−1^ NaCl, 18.38 g.L^−1^ CaCl_2_.2H_2_O, 0.37 g.L^−1^ KCl, and 0.9 g.L^−1^ glucose, pH 5.8) and mixed by inverting the tubes five times. The samples were centrifuged for 5 min at 80 g in a swing out centrifuge (Eppendorf™ 5810R Centrifuge) and the supernatant was removed.

#### Determination of Transfection Efficiencies Using Fluorescence Microscopy

Protoplasts were transfected with 10 or 20 μg pCDB-Cas9-GFP-PDS or pKAR6 in at least three independent experiments. The pKAR6 vector (Robert Blanvillain, unpublished data) (Thomson et al., 2011; Supplementary Table 2) expresses GFP under a 35S promotor and was used as a positive control (PC) for protoplast transfection. Protoplasts transfected without vector (Negative Control 1; NC1) and protoplasts without the addition of both PEG and vector (Negative Control 2; NC2), were used as negative controls. After transfection, 1 mL of 0.5 M mannitol was added to the protoplast pellet and the protoplast suspension was transferred into a 6-well plate and cultured in the dark at 23 ± 2°C on an orbital shaker (30 rpm, 20 h). Next, the protoplast suspension was transferred to an Eppendorf tube, centrifuged for 5 min at 80 g in a swing out centrifuge and supernatant was removed. Twenty μL of the protoplast suspension was transferred to a Bürker chamber and analyzed with a Zeiss AxioImager M2 fluorescence microscope equipped with an Axiocam MRm camera and ZEN software and magnification 200 × (Carl Zeiss MicroImaging, Belgium). Transfection efficiencies were calculated as the ratio of the number of GFP expressing protoplasts (GFP Zeiss filter set 10 (excitation 489 nm, emission 509 nm) to the total number of living protoplasts (based on round shape under bright field microscopy).

#### Regeneration of Transfected Protoplasts

Protoplasts were transfected using 20 μg pCDB-Cas9-PDS in three independent experiments. Subsequent protoplast transfection experiments used a minimum of 10 μg pCDB-Cas9-GAS, pCDB-Cas9-GAO, or pCDB-Cas9-COS. Protoplasts transfected without vector (NC1) and protoplasts without the addition of both PEG and vector (NC2) were used as negative controls. After transfection, 600 μL of regeneration medium (½ Murashige & Skoog macro elements (without NH_4_NO_3_ and KNO_3_) (Murashige and Skoog, 1962) with Heller micro elements (Heller, 1953) and Morel & Wetmore vitamins (Morel and Wetmore, 1951), 18.3 mg.L^−1^ FeNA-EDTA, 750 mg.L^−1^ KCl, 100 mg.L^−1^ inositol, 750 mg.L^−1^ glutamine, 10 g.L^−1^ sucrose, 60 g.L^−1^ mannitol, 0.5 mg.L^−1^ NAA, 0.5 mg.L^−1^ BAP, pH 5.5) was added to the protoplast pellet and the protoplasts were regenerated into plants following the protocol described by Deryckere et al. (2012). After 4 to 5 months, the pCDB-Cas9-GAS, pCDB-Cas9-GAO, pCDB-Cas9-COS transfected shoots and respective control shoots were acclimatized for 4 weeks under a fog tunnel construction with plastic covering (temperature ± 25 °C, 70–80% relative humidity). Afterwards, plantlets were transferred to pots (Ø; 9 cm) and grown in a peat based substrate (1.5 kg.m^−3^ fertilizer: 12N:14P:24K + trace elements, pH 5.0–6.5, EC 450 μS.cm^−1^, Van Israel, Geraardsbergen, Belgium) under greenhouse conditions (temperature ± 20°C, 60–65% relative humidity). The frequency of the albino phenotype of the pCDB-Cas9-PDS transfected plants was calculated by dividing the number of albino plantlets by the total number of regenerated plants, maintained *in vitro*.

### Ploidy Level Analyses

Ploidy levels were analyzed on 442 *in vitro* regenerated plants (pCDB-Cas9-PDS transfected plants and NC1 and NC2 control plants) and 182 acclimatized greenhouse plants (pCDB-Cas9-GAS, pCDB-Cas9-GAO, pCDB-Cas9-COS transfected plants, and NC1 control plants). Approximately 1 cm^2^ of leaf tissue of both the sampled witloof plant and *Pisum sativum*, as an internal standard, were prepared together. The leaf samples were ground with one 3 mm zirconium bead (VWR, Leuven, Belgium) in a 2 mL Eppendorf tube in 250 μL of buffer 1 (0.1 M citric acid monohydrate and 0.5% Tween 20) (Otto, 1997) using a TissueLyser II (Retsch Qiagen, Aartselaar, Belgium) at 20 Hz during 2 min. The ground material was filtered over a 50 μm filter (CellTrics, Sysmex) and stained with 750 μL of buffer 2 [0.4 M Na_2_HPO_4_.12H_2_O and 2 mg.L^−1^ 4′, 6-diamidino-2-phenylindole (DAPI)] (Otto, 1997). Ploidy analysis was performed using a CyFlow Space flow cytometer equipped with a UV Light Emitting Diode (365 nm) (Sysmex, Münster, Germany) and Flomax 2.11 software (Quantum Analysis, Münster, Germany). Ploidy levels were derived from the ratio between the peak position of the sample and the internal standard on the histograms and compared to ratios obtained from the analysis of control witloof plants with known (diploid) ploidy level.

### Molecular Analyses

Genomic DNA of 557 regenerated plants (10 albino pCDB-Cas9-PDS transfected plants and 540 pCDB-Cas9-GAS, pCDB-Cas9-GAO, pCDB-Cas9-COS transfected plants, and NC1 plants) was extracted from ± 50 mg fresh leaf material using a CTAB method (Doyle and Doyle, 1990). Per sample, DNA concentration was measured using the Nanodrop ND1000 (Isogen Lifescience B.V.) and samples were diluted to obtain a final DNA concentration of maximum 40 ng.μl^−1^. Primers were designed for *CiPDS, CiGAS, CiGAO*, and *CiCOS* (Supplementary Table 3) flanking the gRNA target site and the 100–150 bp amplicons were amplified using a highly multiplex (HiPlex) PCR reaction, while attaching sample-specific barcodes. Amplicons from all samples were pooled and ligated to Illumina TruSeq sequencing adapters using the KAPA Hyper prep PCR-free ligation kit according to manufacturer directions (Kapa Biosystems, United States). HiPlex amplification reactions and library preparations were performed by Floodlight Genomics LLC (Knoxville, TN, United States). The libraries were sequenced with 150 PE on a HiSeq3000 instrument (Admera, United States). Forward and reverse reads were merged with PEAR (v0.9.8) (Zhang et al., 2014), sample-specific barcodes were used for sample demultiplexing with custom python scripts and sample-specific barcodes and linker sequences introduced during library preparation were removed. The following steps were performed per sample, and processed in parallel. Reads were sorted per gene by mapping [BWA-MEM with default parameters Li and Durbin, 2009] to the reference gene sequences for *CiPDS, CiGAS, CiGAO*, and *CiCOS*, and the original fastq read files with all HiPlex reads per sample were split into subsets of reads per gene per sample using the readID. The gene-specific amplification primers were removed by trimming the reads with Cutadapt (Martin, 2011) and the remaining sequence window (defined as the entire sequence between the HiPlex primers per gene) was considered as an allele per gene. All unique read sequences, including any potential novel (non-reference) alleles originating from genome editing, were counted per gene per sample. After processing all samples, an integrated table was created listing all read counts per allele per gene per sample across the sample set. Next, the relative allele frequency was calculated as the number of reads per allele per gene per sample divided by the total number of reads per gene per sample. Low frequency alleles were removed using a minimal allele frequency threshold of 2% for analyzing *CiPDS* and 6% for analyzing *CiGAS, CiGAO*, and *CiCOS*. This frequency threshold was calibrated based on empirical observations of the distribution of allele frequencies of alternative (non-reference) sequences in wild type (non-mutated) loci, which were assumed to be derived from PCR artifacts, including sequence jumps; low-frequency sequencing errors, such as base calling errors inherent to Illumina short-read sequencing, read mapping errors, etc. A detailed illustration of the workflow is presented in Supplementary Figure 2. To search for possible insertions of fragments of the transfection vector at the genome-edited loci, any alleles containing a long insertion compared to the reference sequence (>15 nucleotides), and independent of relative allele frequency, were mapped to the pCDB-Cas9 vector. Seven out of 540 samples (1.3%; 6 pCDB-Cas9-GAS and 1 pCDB-Cas9-GAO transfected plants) showed irregular sequencing results and were excluded from the analysis. The overall plant mutation frequency per target gene was calculated by dividing the number of regenerated plants containing at least one observed mutated (non-reference) allele by the total number of regenerated plants (i.e., wild type plants + mutated plants). The gene knockout frequency per target gene was calculated by dividing the number of observed frameshift mutations leading to the premature truncation of the protein, by the total number of regenerated plants (i.e., wild type plants + mutated plants).

### *CiPDS* Copy Number Analysis With ddPCR

Droplet digital PCR (ddPCR) was used to quantify the copy number of *CiPDS* in a diploid “Van Hamme” witloof plant according to the protocol described in Desmet et al. (2020) with some minor assay-specific modifications. Two reference genes were selected: the single copy gene *PP2AA3* and *UBQ10*, present in a double amount of copies (Delporte et al., 2015); primers are described in Supplementary Table 3. For the amplification of the *CiPDS*, primer11 and primer12 (Supplementary Table 3) were designed in the same region as the primers used for HiPlex amplicon sequencing of the *CiPDS* target region. Four DNA samples of Van Hamme (1 μg) were digested with EcoRI (10U) and MspI (50U). Ten ng of the digest was used as input material for ddPCR and each sample, including no template controls (NTC), was analyzed in duplicate on the QX200™ (BioRad). Annealing temperature of the PCR was 56°C for these assays and Quantasoft version 1.7.4.0917 (Bio-Rad) was used for calculating concentrations (copies.μL^−1^). Haploid *CiPDS* copy number was calculated as [*CiPDS*] / [reference gene] X 1 or 2 (for *PP2AA3* or *UBQ10*, respectively). Finally mean (+/– stdev) copy number was calculated over both reference genes and all 4 samples.

## Results

### A CRISPR/Cas9-Vector Based Protoplast Transfection and Regeneration Protocol

Protoplasts of two witloof varieties (“Van Hamme” and “Topmodel”) were transfected with 10 or 20 μg of the pCDB-Cas9-GFP-PDS vector to test the effect of vector concentration on protoplast transfection efficiency (Table 1). Protoplast transfection efficiencies with pCDB-Cas9-GFP-PDS were between 20 and 26% in both varieties. For “Van Hamme” protoplasts, this was similar to the transfection efficiency when using the positive control vector pKAR6, while in “Topmodel” protoplasts the transfection efficiency with pKAR6 was markedly higher (44.2%). No GFP fluorescence was observed in any of the negative control treatments. Overall, these results show that 10 μg of vector DNA is sufficient to obtain efficient protoplast transfection.

**Table 1:** Fluorescence microscopy image of witloof “Van Hamme” protoplasts transfected with pCDB-Cas9-GFP-PDS (left panel) and transfection efficiencies with the pCDB-Cas9-GFP and pKAR6 vector (right panel).

**Fluorescence microscopy image**	**Variety**	**Vector**	**DNA**	**Efficiency (%)**
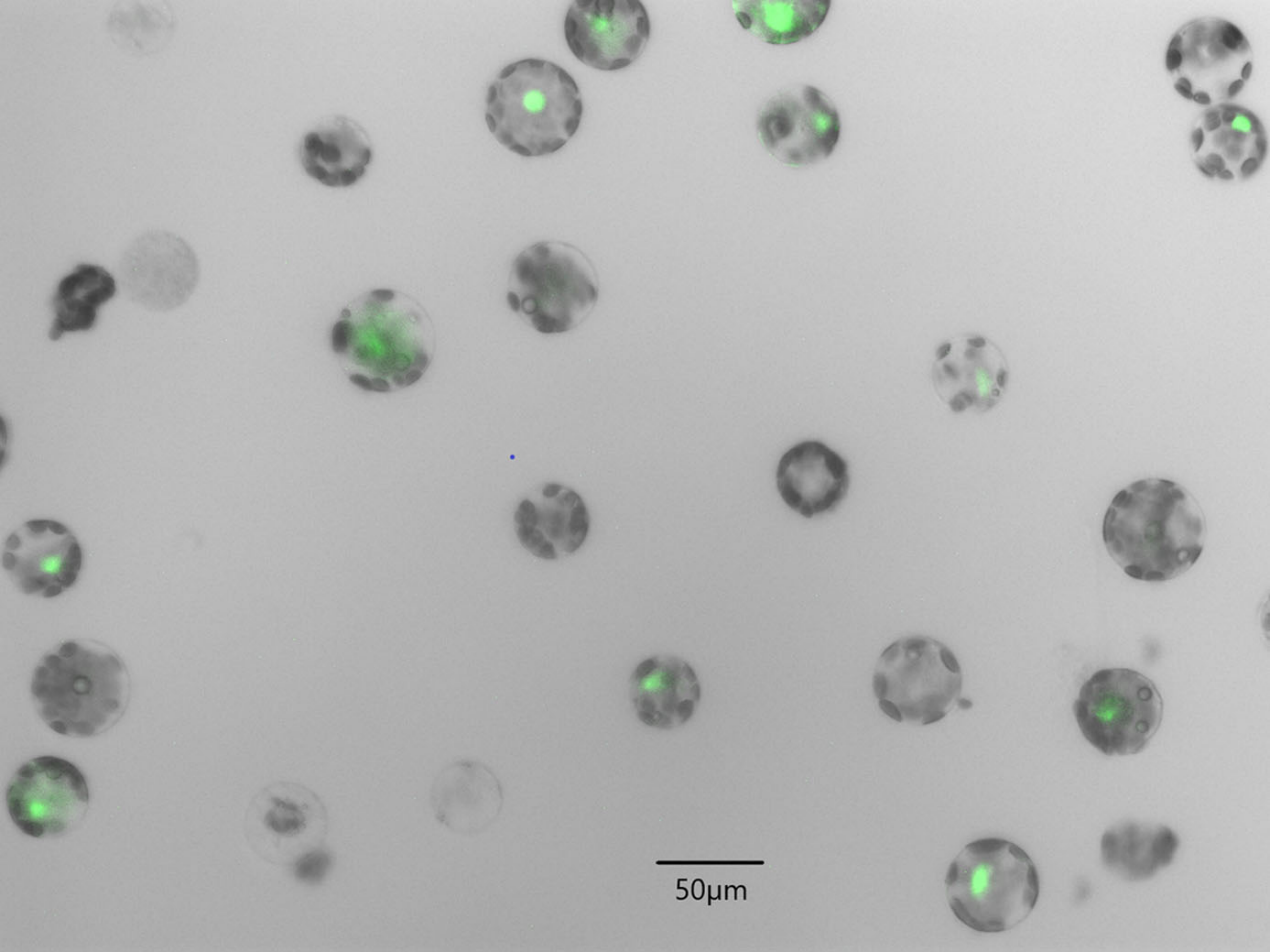	Witloof “Van Hamme”	pKAR6	10 μg	22.9 ± 2.6
		pCDB-Cas9-GFP-PDS	10 μg	20.5 ± 4.2
		pCDB-Cas9-GFP-PDS	20 μg	24.0 ± 5.9
		NC1	–	0.0
		NC2	–	0.0
	Witloof “Topmodel”	pKAR6	10 μg	44.2 ± 4.0
		pCDB-Cas9-GFP-PDS	10 μg	20.7 ± 7.2
		pCDB-Cas9-GFP-PDS	20 μg	26.0 ± 1.9
		NC1	–	0.0
		NC2	–	0.0

*NC1, protoplasts transfected with PEG but without vector; NC2, protoplasts without addition of both PEG and vector (mean +/– stdev; n = 3–5)*.

Witloof “Van Hamme” was selected for subsequent transfection and regeneration experiments because of its higher regeneration capacity (data not shown) and more consistent transfection efficiencies (Table 1). Protoplasts were transfected with pCDB-Cas9-PDS to induce mutations in *CiPDS* which can result in albino plantlets (Zhang et al., 2017). Protoplast transfection with pCDB-Cas9-PDS and subsequent regeneration yielded a total of 186 regenerated plants over three independent experiments, among which we observed a total of 55 albino plantlets during *in vitro* culture. The frequency of observed albino phenotypes to the total number of regenerated plants varied from 21 to 27% (mean 23 ± 3%; Table 2) and no albino plantlets were observed in the 355 negative control regenerated plants.

**Table 2:** Regeneration of witloof protoplasts and frequency of albino phenotypes.

	**Exp**.	**# Green**	**# Albino**	**Frequency (%)**
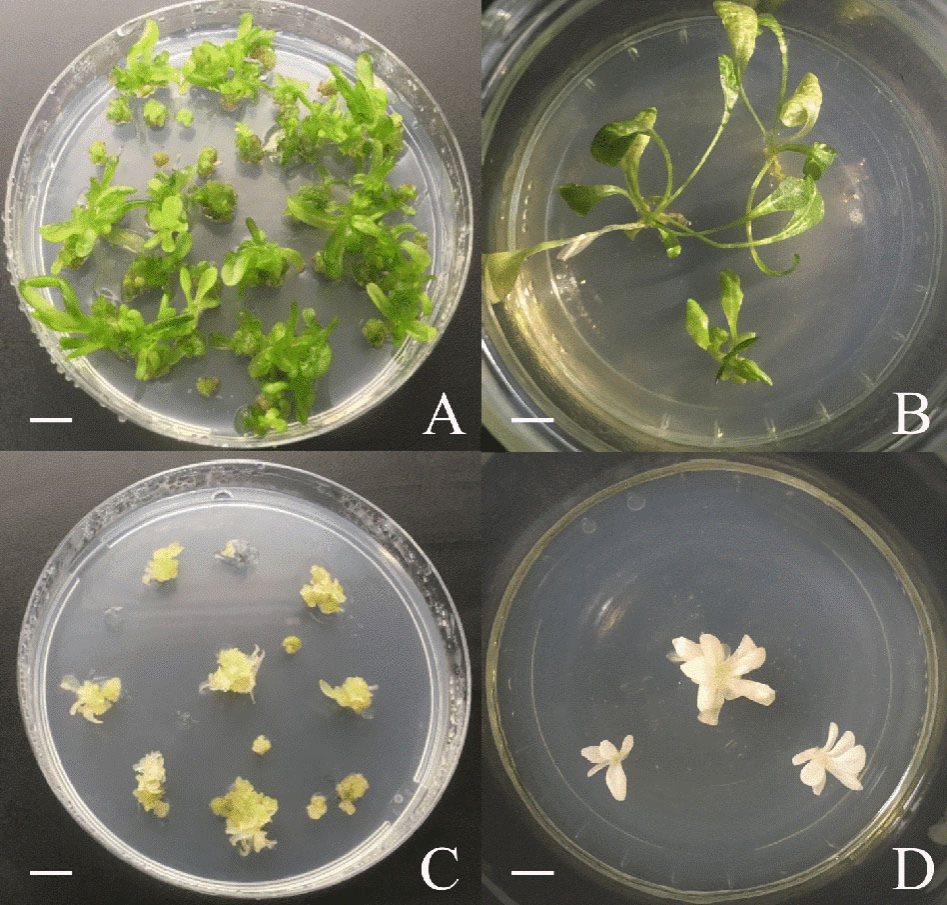	1	30	8	21.0
	2	45	17	27.4
	3	111	30	21.3
	Total	186	55	**23.3** **±** **3.0**
	NC1	127	0	0.0
	NC2	228	0	0.0

*Left Panel: **(A)** Wild-type (WT) shoots emerging from callus, **(B)** WT plants, **(C)** CiPDS mutated albino shoots, and **(D)** CiPDS mutated albino plants. Scale bar, 1 cm. Right Panel: Witloof “Van Hamme” plants were transfected with 20 μg pCDB-Cas9-PDS. Frequency of albino phenotypes are given per experiment (1, 2, 3); Frequency is given as mean +/– stdev across the three experiments (n = 3, in bold). NC1, protoplasts transfected with PEG but without vector; NC2, protoplasts without addition of both PEG and vector*.

HiPlex amplicon sequencing was used to determine the genomic DNA sequence at the *CiPDS* target loci of ten albino plantlets. All other 533 regenerated plants (see description of *CiGAS, CiGAO*, and *CiCOS* targeted plants below in 3.2) were used as controls for *CiPDS* sequencing. In all plants (control and albino), the *CiPDS* primers consistently amplified two different sequence variants, differentiated by a single SNP [localized 39 nucleotides upstream from the gRNA target site (Supplementary Table 4)]. The ten albino plants showed different combinations of two mutated alleles derived from *CiPDS* reference locus 1 (Tables 3, 4, listed as *CiPDS* locus 1) and all seven unique mutated alleles carried frameshift mutations that lead to premature termination and functional knockout of the encoded protein. These observations can be explained by the hypothesis that these alleles are derived from a locus that is homozygous in diploid wild type plants and can give rise to two alternative alleles upon genome-editing. The second reference locus (Tables 3, 4, listed as *CiPDS* locus 2) was also edited, giving rise to a total of 14 unique mutated alleles. The number of unique mutations varied between the 10 albino plants and, strikingly, gave rise to up to six different mutated alleles in a single diploid plant, suggesting three additional paralogous copies of the gRNA target site and flanking regions (Plant A4, Table 3). All mutations were positioned at the predicted Cas9 cut site (Table 4), consistent with DSB followed by imperfect NHEJ repair. The alleles with large insertions contained relatively large fragments (21, 26, 70, and 87 nucleotides) of the transfection vector. The copy number of the *CiPDS* gRNA target region was estimated using droplet digital PCR (ddPCR) and resulted in the presence of at least four copies (4.54 +/− 0.18; Supplementary Table 5). Taken together, the observation of up to eight different mutated *CiPDS* alleles in a single diploid plant can be explained by the presence of at least four paralogous copies of the *CiPDS* gRNA target site in plant variety “Van Hamme,” in line with the ddPCR results. Our data further show that multiple loci may be edited simultaneously by a common gRNA targeting a conserved sequence and their parallel detection was possible with the common HiPlex *CiPDS* primers.

**Table 3:** Genotype of the ten albino plantlets and their corresponding ploidy level.

**Plant**	**Ploidy**	***CiPDS* Locus 1**	***CiPDS* Locus 2**
A1	Diploid	I1A/I1T	WT/I1T/D10I88
A2	Diploid	I1A/I1T	WT/I1T/D10I88
A3	Diploid	I1A/I1T	WT/I1T/D10I88
A4	Diploid	I1C/I1T	D9/D9I2/D3/D2/WT/I1A
A5	Diploid	D8I6/I1T	D10/D2/D9I30/D1I25
A6	Diploid	D5/I1T	D13/D3/WT/I70
A7	Tetraploid	D2/I1A	WT
A8	Tetraploid	D2/I1A	WT
A9	Tetraploid	I1T	WT/I1G
A10	Tetraploid	D14/I1T	D3/D2/D1/WT/I1T

*I, Insertion; D, Deletion; WT, Wild Type. Mutation types and plant genotypes with mutations leading to early stop codons, truncating the protein translation are underlined*.

**Table 4:**
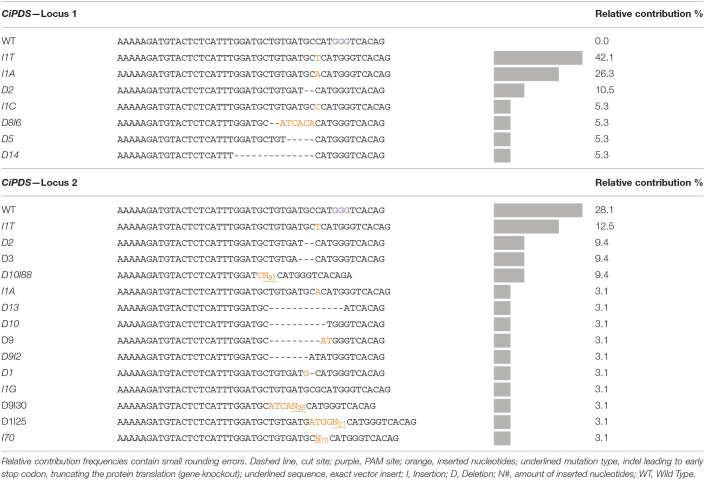
Overview of the mutation types and relative contribution of each mutation type in the ten mutated albino plants.

As plant regeneration from protoplasts may lead to changes in ploidy level (Larkin and Scowcroft, 1981), we scored the frequency of ploidy changes under our conditions of protoplast transfection and regeneration. The ploidy level of 442 *in vitro* regenerated plants (both pCDB-Cas9-PDS transfected plants and NC1 and NC2 control plants), were analyzed by flow cytometry, showing 77.2% diploid plants, 21.5% tetraploid plants and the remaining 1.3% consisted of haploids, hexaploids and mixoploids.

Taken together, these results show that our protocol for genome editing based on transfection and regeneration of witloof protoplasts without the use of typical plant selection markers (e.g., kanamycin, hygromycin, *bar* gene), yielded about 23% albino plantlets amongst all regenerated plants (Table 2).

### CRISPR/Cas9 Induced Mutations in the SL Biosynthesis Pathway Genes GAS, GAO, or COS

Our genome editing protocol was then used to target the *CiGAS, CiGAO*, and *CiCOS* genes, known to be involved in the SL biosynthesis pathway (Bouwmeester, 2002; Liu et al., 2011). Witloof “Van Hamme” protoplasts were transfected with either the pCDB-Cas9-GAS, pCDB-Cas9-GAO, or pCDB-Cas9-COS vectors. A total of 533 plants were regenerated and genetically characterized at all target loci, including 11 control plants (NC1), 374 plants transfected with pCDB-Cas9-GAS, 89 plants transfected with pCDB-Cas9-GAO and 59 plants transfected with pCDB-Cas9-COS. The ploidy level was analyzed for 182 regenerated plants showing 80.8% diploid plants, 18.1% tetraploid plants and 1.1% consisting of haploids, hexaploids, and mixoploids. Out of the 522 regenerated plants after transfection, mutation analysis revealed 324 wild type plants, 137 plants with a monoallelic mutation (one reference allele and one mutated allele), 5 plants with a single type of mutated allele (but no reference allele detected) and 56 plants with biallelic mutations (two different mutated alleles) (Table 5). Furthermore, 19 plants contained indels resulting in a premature truncation of the protein (presumably gene knockout) in all observed alleles per plant (Table 5). This resulted in an overall plant mutation frequency of 37.9% and gene knockout frequency of 3.6%. More specifically, in pCDB-Cas9-GAS transfected plants, the plant mutation frequency was 27.3% (Table 5) with a total of five mutated plant genotypes (Table 6). The pCDB-Cas9-GAO transfected plants showed a plant mutation frequency of 42.7% (Table 5) and a total of 18 mutated plant genotypes (Table 6). In pCDB-Cas9-COS transfected plants, the plant mutation frequency was 98.3% (Table 5) and only one mutated plant genotype was observed (M24) (Table 6). Additionally, nine *CiGAO* mutated plant genotypes (M7, M9, M12, M13, M17, M18, M20, M21, and M23) showed premature truncation of the CiGAO protein due to a frameshift mutation in all observed alleles, resulting in a *CiGAO* gene knockout frequency of 21.4%, while no homozygous knockouts were created in *CiGAS* or *CiCOS* mutated plants (Table 6).

**Table 5:** Overview of the mutation events in the pCDB-Cas9-GAS, pCDB-Cas9-GAO, and pCDB-Cas9-COS protoplast transfected and regenerated plants.

**Genes**	**GAS**	**GAO**	**COS**	**Total**	**Control**
Total number of regenerants	374	89	59	522	11
WT	272	51	1	324	11
WT/Indel	71	8	58	137	0
Indel (KO)	0 (0)	5 (3)	0 (0)	5 (3)	0 (0)
Indel/Indel (KO/KO)	31 (0)	25 (16)	0 (0)	56 (16)	0 (0)
Plant mutation frequency^a^	27.3%	42.7%	98.3%	37.9%	0.0%
Gene knockout frequency^b^	0.0%	21.4%	0.0%	3.6%	0.0%

*WT, Wild Type plant; WT/Indel, Monoallelic mutation; Indel, Single observed mutation; Indel/Indel, Biallelic mutation; KO, Gene knockout (indel leading to early stop codon, truncating the protein translation).*

a *Calculated by dividing the sum of WT/Indel, Indel and Indel/Indel by the total number of regenerants: (WT/Indel + Indel + Indel/Indel)/TotReg.*

b *Calculated by dividing the sum of KO and KO/KO by the total number of regenerants: (KO + KO/KO)/TotReg*.

**Table 6:** Overview and number of *CiGAS, CiGAO* and *CiCOS* mutated plant genotypes.

**Target gene**	**Plant**	**Plant genotype**	**# plants**
CiGAS	M1	WT/D11	25
	M2	WT/D10	12
	M3	WT/D7	19
	M4	WT/D6	15
	M5	D6^*^/D8I3	31
CiGAO	M6	D27/D10	1
	M7	D26	1
	M8	D12/D12^*^	2
	M9	D4	1
	M10	D4/D2I92	1
	M11	D3/D14	5
	M12	D2/I47	2
	M13	D1/D4	2
	M14	WT/D2	2
	M15	WT/I1T	6
	M16	D12	1
	M17	I1T/D17	6
	M18	I1A/D13	1
	M19	I1T/D3	1
	M20	I1T/D2	2
	M21	I1A	1
	M22	I30	1
	M23	D12I55/I104	2
CiCOS	M24	WT/I1A	58

*WT/Indel, Heterozygous mutation; Indel, Single observed mutation; Indel/Indel, Compound heterozygous mutation, I, Insertion, D, Deletion,*

* *, different mutation type; WT, Wild Type. Mutation types and plant genotypes with mutations leading to early stop codons, truncating the protein translation are underlined*.

Table 7 gives a detailed overview of the detected mutation types and their relative contribution within the *CiGAS, CiGAO*, and *CiCOS* mutated plants. The *CiGAS* amplicon reads revealed six different mutation types across all *CiGAS* mutated plants (Table 7). The *CiGAO* amplicon reads revealed a total of 20 different mutation types across all *CiGAO* mutated plants (Table 7). Notably, five of the *CiGAO* mutated plants showed, in at least one of the alleles, an insertion which was part of the Cas9 vector with fragment lengths of 30, 43, 47, 90, and 104 nucleotides. The *CiCOS* amplicon reads revealed a mutation type containing a single “A” nucleotide insertion across all *CiCOS* mutated plants (Table 7). No mutations were detected among the 11 control plants and in the observed loci that were not targeted by the gRNAs. These results show that our protocol for genome editing based on transfection and regeneration of witloof protoplasts, can create a variety of mutation types and mutated plant genotypes. Furthermore, gene knockouts can be created that could be able to change the SL metabolite content and result in altered bitterness.

**Table 7:**
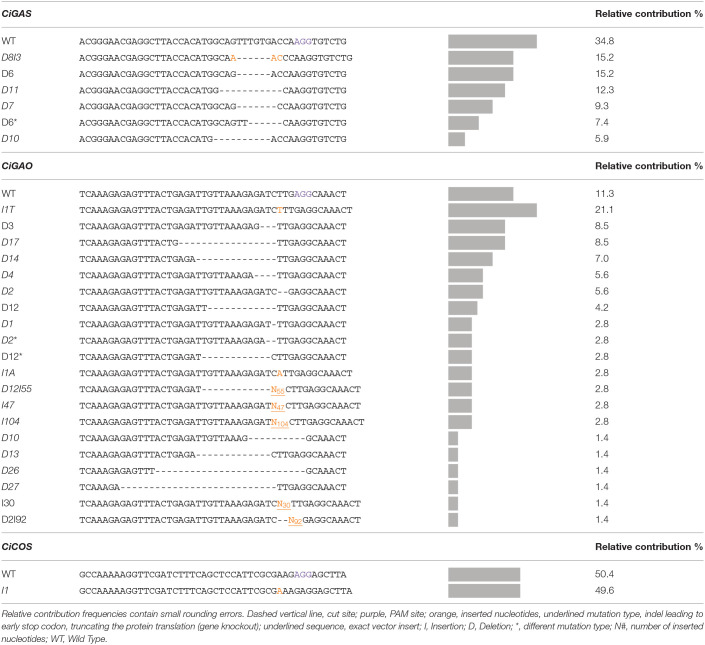
Overview of the mutation types and relative contribution of each mutation type in the plants transfected with pCDB-Cas9 vector targeting *CiGAS, CiGAO*, and *CiCOS*.

## Discussion

CRISPR/Cas9 genome editing is a powerful tool for both gene function research and plant breeding and was already successfully applied on several crops such as maize, rice, tomato, among others (Manghwar et al., 2019; Zhang et al., 2019). We evaluated CRISPR/Cas9 in protoplast transfection and regeneration experiments for witloof and analyzed protoplast transfection efficiencies, CRISPR/Cas9 mutation efficiencies, mutation spectra and *in vitro* tissue culture observations (ploidy changes and clonal propagation).

Firstly, we confirmed CRISPR/Cas9 vector expression in the protoplasts using PEG transfection and a CRISPR/Cas9 vector containing a GFP marker in two witloof varieties “Van Hamme” and “Topmodel.” Protoplast transfections resulted in transfection efficiencies around 20 to 26%. Previous reports have shown variable PEG mediated protoplast transfection efficiencies, for instance, in cherry (33.4%) (Yao et al., 2016), orchid (41.7%) (Li et al., 2018), rice (53–75%) (Zhang et al., 2011) and poinsettia (>70%). Those studies showed the impact of multiple parameters such as species, plant tissue, PEG concentration, vector size, vector concentration and transfection duration. In addition, those studies were aimed at achieving maximal transfection efficiencies, without taking into consideration long-term protoplast viability and protoplast regeneration capacity. In our study, we obtained 20 to 26% transfection efficiency while still obtaining successful regeneration. Hence, we established an efficient transfection protocol in witloof protoplasts, which makes further applications for witloof genome editing possible.

The mutation efficiency in witloof using CRISPR/Cas9 was initially estimated by targeting the *CiPDS* gene using a single gRNA and without the use of selective media. The *PDS* gene is often used as a target during the development of genome editing techniques as it allows direct visual screening for albino phenotypes resulting from CRISPR/Cas9 induced mutations. The *PDS* gene has been targeted in many species such as wheat and tobacco (Upadhyay et al., 2013), rice (Shan et al., 2013), poplar (Fan et al., 2015), apple (Nishitani et al., 2016), watermelon (Tian et al., 2017), cassava (Odipio et al., 2017), banana (Kaur et al., 2018; Naim et al., 2018), and recently also chicory (Bernard et al., 2019). In our study, a CRISPR/Cas9 vector targeting the first exon of *CiPDS* was delivered into witloof protoplasts and resulted in albino plants in 23% of the regenerated plants. DdPCR analysis revealed at least four paralogous copies of the *CiPDS* gRNA target sequence, while HiPlex amplicon sequencing analysis showed two types of *CiPDS* gRNA flanking sequences in the “Van Hamme” genome, which can be distinguished based on a SNP 39 nucleotides away from the gRNA target site. All tested plants with an albino phenotype carried a knockout mutation in all observed alleles in locus 1 of the *CiPDS* gene. The other loci displayed variable numbers of mutated alleles, reaching a total of up to eight different mutation types in the *CiPDS* alleles of an albino individual. Taken together, these data show that a single gRNA that binds a conserved sequence can effectively and simultaneously induce mutations at multiple genomic loci. PEG-mediated protoplast transfection targeting *PDS* in other species showed highly variable mutation frequencies, probably because of numerous experimental parameters, such as gene target, target design, species transfection and regeneration ability and ploidy level. For instance, the reported mutation frequency in the *PDS* gene ranged from 1.1 to 5.6% in PEG-transfected *Arabidopsis* protoplasts and was around 37% in tobacco protoplasts (Li et al., 2013). Additionally, parameters related to mutation analysis techniques, such as mutation detection techniques, mutation threshold values and mutation frequency calculations, can also play a role in the reported variability of mutation frequencies. For instance, a mutation frequency of 6.6% was reported in bamboo protoplasts using band intensity calculations of gel electrophoresis images, while a mutation frequency of 12.5% was reported using NGS sequencing and read depth analysis (Lin et al., 2018). Furthermore, protoplasts of tetraploid potato yielded mutations in all four alleles of the *GBSS* gene in up to 2% of regenerated lines, whereas 2–12% of regenerated lines showed mutations in at least one allele of the gene (Andersson et al., 2017). Therefore, comparison of mutation efficiencies should be based on the same quantification analysis. We have further reported our mutation efficiencies in terms of (1) plant mutation frequency to analyze the efficiency of obtaining plants with single, monoallelic, and biallelic mutations, and (2) gene knockout frequency to analyze the efficiency of obtaining plants with a knockout of all observed alleles. Furthermore, these mutation efficiencies can be of interest for gene function analysis and plant breeding purposes. Acceptable mutation efficiencies thus depend on plant species and the objective of the CRISPR/Cas9 genome editing technique. In our research, we demonstrated that genome editing in witloof protoplasts is promising, with transfection rates of at least 20% and frequencies of the albino phenotype of at least 23% which is more than sufficient for subsequent screening and analysis of mutated plant lines without the use of stable transformation and typical plant selection markers.

To further demonstrate the potential of our genome editing approach, three previously described SL biosynthesis pathway genes were targeted using our CRISPR/Cas9 vectors. Characterization of the witloof mutated plants revealed single, monoallelic, and biallelic mutations. Interestingly, in *CiGAO* mutated plants, many different mutation types were observed and 21.4% of the *CiGAO* transfected plants had a mutation in all observed alleles leading to a premature stop codon (knockout). These plants containing knockout mutations in all observed alleles are also the ideal material for studying gene function. Furthermore, self-fertilization of the plants containing a heterozygous knockout mutation (WT/KO) could also generate a homozygous knockout mutation (KO/KO) after Mendelian segregation in the progeny. Further research and creation of next generation plants through self-pollination will provide information about the segregation in the progeny and to verify the success rate of creating these homozygous knockout plants. Overall mutation screening of the 522 transfected greenhouse plants revealed a plant mutation frequency of 38% containing a wide spectrum of mutation types. Mostly a single deletion, insertion, or nucleotide substitution was induced, although sometimes a combination of deletion and insertion was observed. In case the Cas9 enzyme produces a DSB at the target site, a deletion can be followed by an insertion (e.g., D8I3, Table 7). This is supported by previous research on the repair of DSBs in plants, which shows different combinations can occur during the repair process (Puchta, 2005). However, most mutation analysis reports, typically only list the difference in total sequence length (expressed in number of nucleotides) at the mutated target site compared to the corresponding reference sequence length. We also observed instances of CRISPR/Cas9 DNA vector fragment insertions at the site of the DSBs in the genome of mutated plants. Once the vectors are introduced in the protoplasts, they are digested by endogenous nucleases and yield fragments that can be integrated in the plant genome. This phenomenon has previously been reported, amongst others, in potato (Andersson et al., 2017), tobacco (Lin et al., 2018), and chicory (Bernard et al., 2019). Generally, HiPlex amplicon sequencing allows to screen for vector fragment inserts at the site of the DSBs, but large indels may not be detected if it affects the primer binding site. Alternatively, size-exclusion during library preparation and sequencing also creates a bias against amplicons with a substantial change in length after mutation, resulting from relatively large insertions (>150 bp) or deletions (>90 bp) between the primer binding sites. Further mutation analysis revealed that the number of produced mutation types seems to be linked to the target sequence site. Previously, it has been suggested that the variability of mutation types could also be linked to the intrinsic DNA repair mechanism of the species, transformation method and/or culture conditions (Allen et al., 2019). However, as the target sequence site was the only variable parameter in our research, we can determine the target sequence site as an influencing factor in creating this variable mutation spectrum. This has already been described by Shen et al. (2018) and Liu et al. (2020), implementing a computational method that predicts DNA repair outcomes at DSBs induced by CRISPR/Cas9 resulting from NHEJ. However, using the online computational method inDelphi (https://indelphi.giffordlab.mit.edu/) (Shen et al., 2018) on our target genes, suggested different mutation spectra and contributions. As the model was trained on mammalian cell types, it is not expected to generalize well to bacteria, plants, and non-mammalian eukaryotes.

Working with *in vitro* tissue culture comprising protoplast transfection and regeneration, questions regarding ploidy level changes (Larkin and Scowcroft, 1981) and plant clones arise. Therefore, we analyzed ploidy level changes during protoplast regeneration and observed the formation of around 20 % tetraploids during pCDB-Cas9-PDS and pCDB-Cas9-GAS, pCDB-Cas9-GAO, and pCDB-Cas9-COS protoplast transfection and regeneration experiments. Hereby, we only observed up to two different mutated alleles in *CiGAS, CiGAO*, and *CiCOS* mutated plants, hypothesizing that ploidy level changes occur after the CRISPR/Cas9 mutation event. Ultimately, using CRISPR-Cas9 mutated plants in witloof breeding requires to screen and select for diploid regenerated plants, and it is important to monitor ploidy changes in an early stage after plant regeneration. We thereafter analyzed the frequency with which clonal lines are obtained from callus. Using our mutation data, we observed the presence of a high number (18) of unique mutated plant genotypes in all (38) *CiGAO* mutated plants (Table 6), suggesting a low occurrence of clonal plant lines. Nevertheless, two plant genotypes (M12 and M23; Table 6) carrying the same vector fragment insert (e.g., the exact same sequence of 47 nucleotides), illustrate that in our experiments occasionally clonal lines were obtained from individual calli. As it is unlikely that the same vector fragment insert was introduced into the genome of more than one independent protoplast, we speculate that some separately analyzed plants actually originate from the same transfected and mutated protoplast, resulting in multiple plant clonal genotypes. While during regeneration, each callus can produce multiple shoots, selection of only one shoot per callus will reduce the number of clonal plant genotypes. However, the presence of a high number of plants containing the same mutated plant genotype (e.g., M24, Table 6) could also be the result of a preferred DNA repair outcome at DSBs induced by CRISPR/Cas9 resulting from NHEJ. The high mutation frequency and low mutation variation observed for pCAS9-COS transfected plants (M24) could thus result from the preferred “A” nucleotide insertion during NHEJ, possibly combined with some level of clonal propagation.

We have shown that the CRISPR/Cas9 technology is very valuable to induce targeted mutations in four genes of witloof. To further implement a DNA-free genome editing technique, it would be interesting to use pre-assembled ribonucleoprotein complexes (RNPs) instead of vector DNA to deliver Cas9/gRNA into *Cichorium* protoplasts. RNPs have been used in *Arabidopsis*, tobacco, lettuce, and rice protoplasts, through PEG-mediated transfection, using the same conditions as with vector DNA transfection, leading to higher mutation frequencies and eliminating the chance of vector fragment insertions (Wook Woo et al., 2015). In conclusion, CRISPR/Cas9 genome editing is of significant importance for future witloof breeding as it comprises a powerful tool for investigating gene functions and altering agronomical traits in commercially interesting witloof varieties.

## Author Contributions

CD, KV, TE, TR, ED, TJ, and AG: study conception. TE and CD: design of experiments and protoplast assays. TJ and CD: vector design. CD: production of gene edited plants and wrote the manuscript. TR and CD: NGS data analysis and interpretation. KV and CD: overall overview of experiments. KV and TR: revised the manuscript drafts. All authors contributed to the manuscript revision, read, and approved the submitted version.

## Conflict of Interest

The authors declare that the research was conducted in the absence of any commercial or financial relationships that could be construed as a potential conflict of interest.
