# Supplementary material for: Establishment of CRISPR/Cas9 Genome Editing in Witloof (Cichorium intybus var. foliosum)
Source: Front Genome Ed. 2020 Dec 21;2:604876. doi: 10.3389/fgeed.2020.604876 (PMC8525355; doi:10.3389/fgeed.2020.604876)
Supplement: Supplementary file 1 [file Data_Sheet_1.docx]

Supplementary Material

# Supplementary Figures and Tables

## Supplementary Figures

| **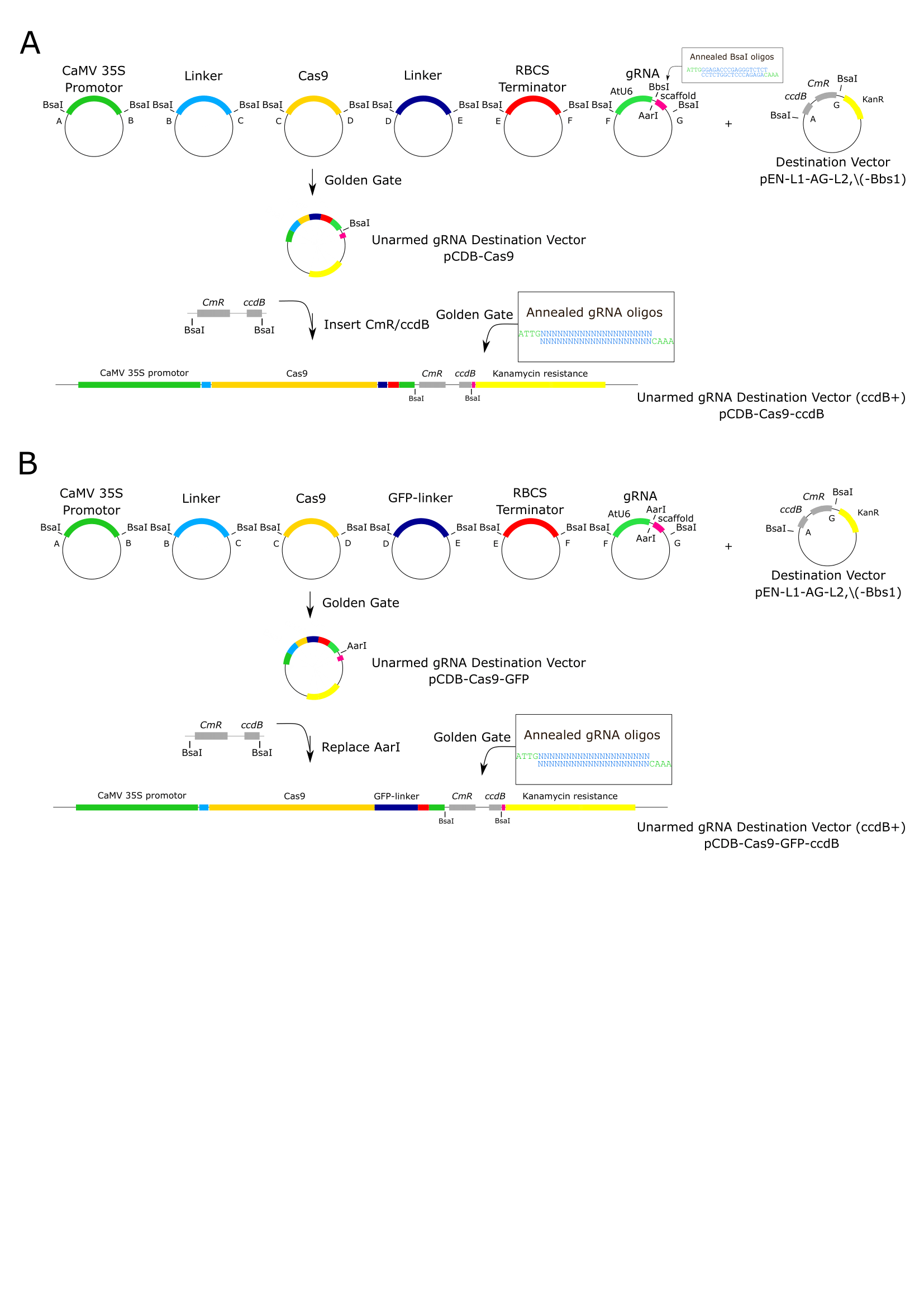**  **Supplementary Figure 1**. Construction of (A) the Cas9 expression vector and (B) the Cas9-GFP expression vector. |
| --- |

| **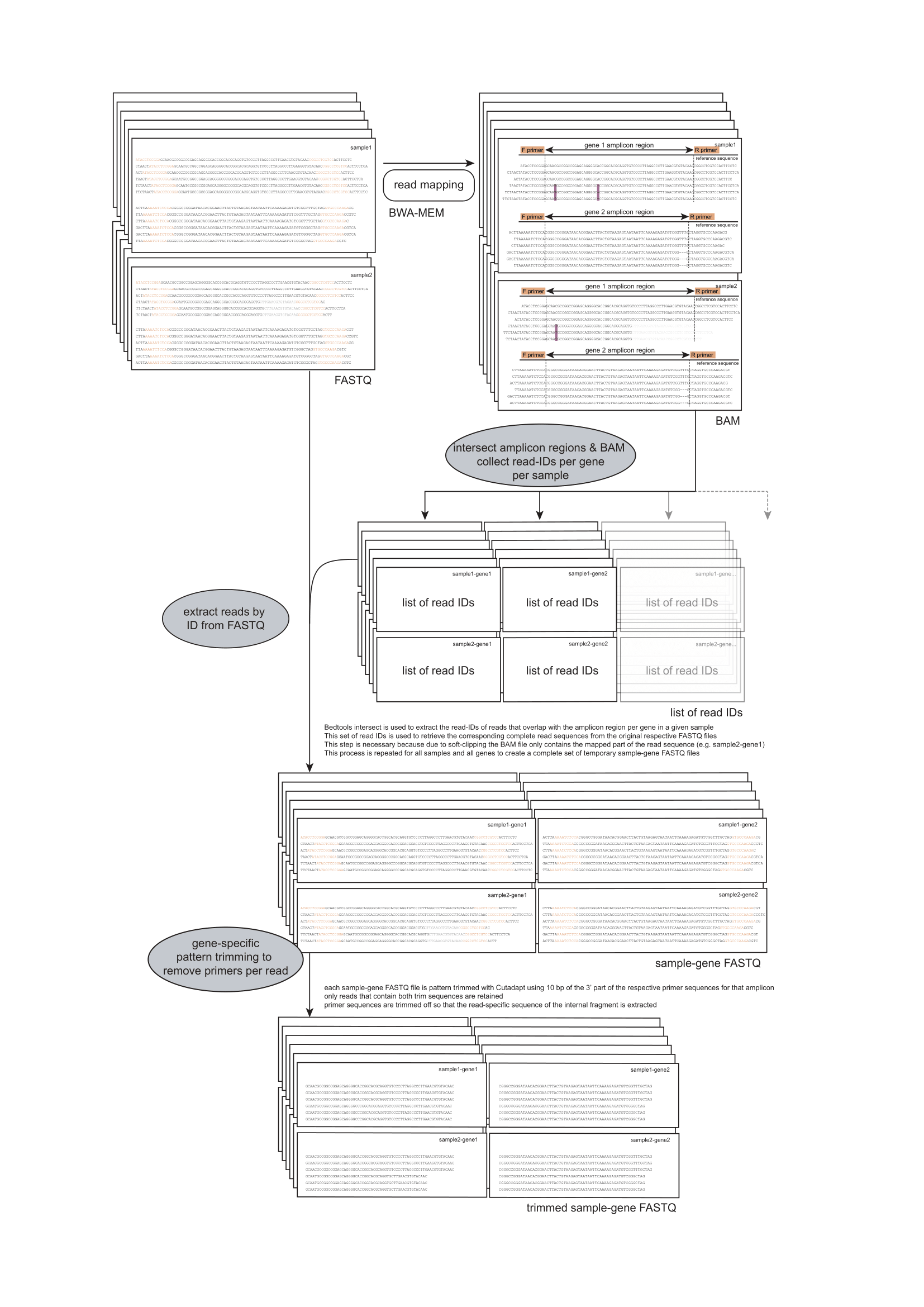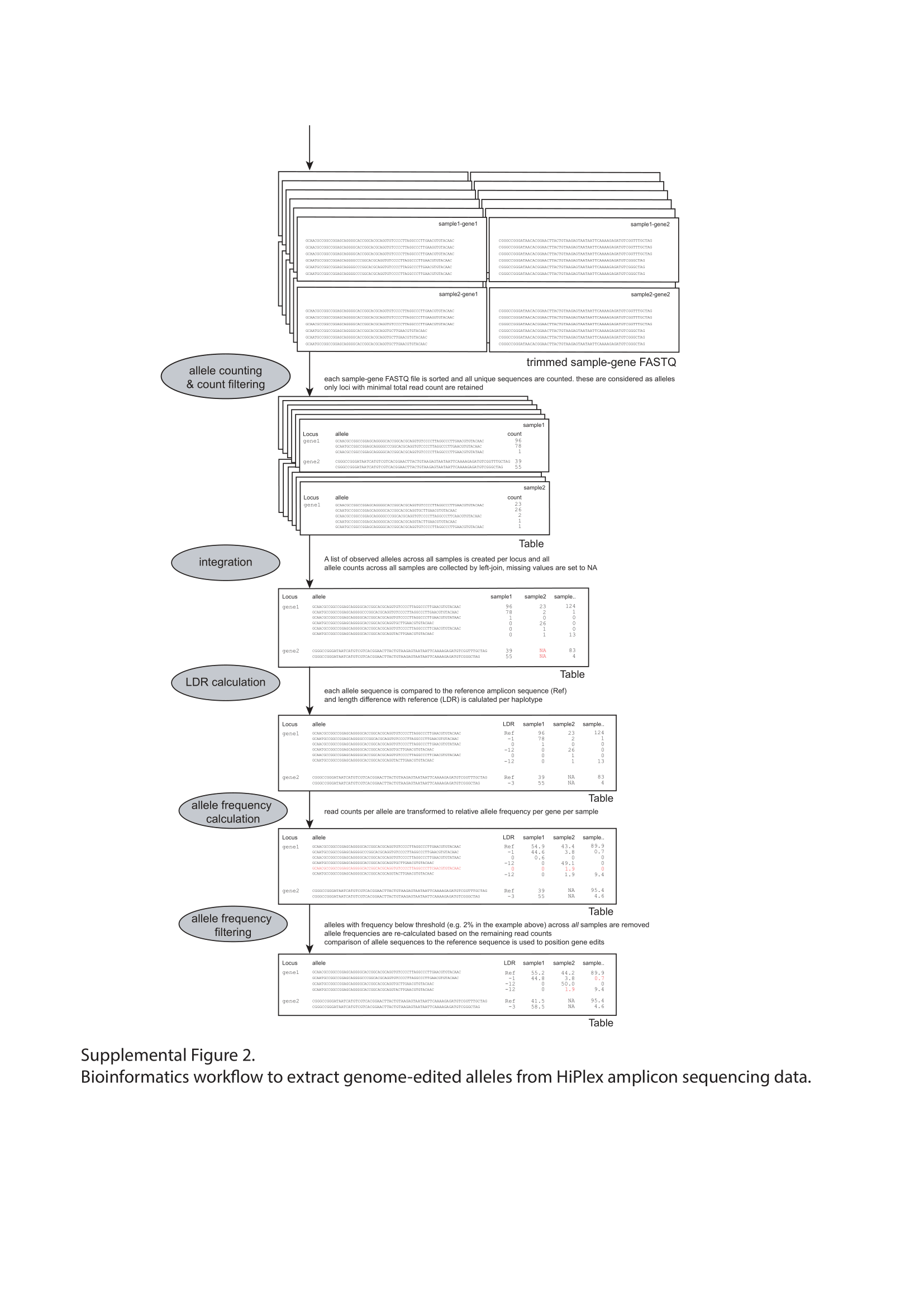**  **Supplementary Figure 2**. Bioinformatics workflow to extract genome-edited alleles from HiPlex amplicon sequencing data |
| --- |

## Supplementary Tables

Supplementary Table 1. Oligonucleotide and primer sequences. Underlined = N20 guide RNA sequence, *Italic* = vector overhangs

| **Oligo/Primer** | **Sequence** | **Purpose** |
| --- | --- | --- |
| Oligo1 | 5’-*ATTG*TTTGGATGCTGTGATGCCAT-3’ | PDS oligo F |
| Oligo2 | 5’-*AAAC*ATGGCATCACAGCATCCAAA-3’ | PDS oligo R |
| Oligo3 | 5’-*ATTG*CACATGGCAGTTTGTGACCA-3’ | GAS oligo F |
| Oligo4 | 5’-*AAAC*TGGTCACAAACTGCCATGTG-3’ | GAS oligo R |
| Oligo5 | 5’-*ATTG*AGATTGTTAAAGAGATCTTG-3’ | GAO oligo F |
| Oligo6 | 5’-*AAAC*CAAGATCTCTTTAACAATCT-3’ | GAO oligo R |
| Oligo7 | 5’-*ATTG*TTTCAGCTCCATTCGCGAAG-3’ | COS oligo F |
| Oligo8 | 5’-*AAAC*CTTCGCGAATGGAGCTGAAA-3’ | COS oligo R |
| Oligo9 | 5’-*ATTG*GGAGACCCGAGGGTCTCT-3’ | BsaI insert |
| Oligo10 | 5’-*AAAC*AGAGACCCTCGGGTCTCC-3’ | BsaI insert |
| Primer20 | 5’-TCCCAGGATTAGAATGATTAGG-3’ | pCas9_F |
| Primer24 | 5’-CCAGGAAACAGCTATGACCAT-3’ | pCas9_R |
| BbsIApaI_S | 5’-GGCCCAGTCATCCGACTGAGCCTTTCGTTTTATTTGATGCCTGGCAGTTCCCTACTCTCGCGTTAACGCTAGCATGGATGTTTTCCCAGTCACGACGTTGTAAAACGACGGCCAGTCTTAAGCTCGGGCC-3’ | BbsI mutated insert |
| Bbs1Apa1_AS | 5’-CGAGCTTAAGACTGGCCGTCGTTTTACAACGTCGTGACTGGGAAAACATCCATGCTAGCGTTAACGCGAGAGTAGGGAACTGCCAGGCATCAAATAAAACGAAAGGCTCAGTCGGATGACTG-3’ | BbsI mutated insert |

Supplementary Table 2. Vector overview. ^a^The cloning sites are four-nucleotides overhangs for Golden Gate cloning (according to Lampropoulos et al. 2013)

| **Full Name** | **Type** | **Cloning sites^a^** | **Bacterial Selection** | **Length** | **Reference** | **Plasmid Deposit** |
| --- | --- | --- | --- | --- | --- | --- |
| **Golden gate plant promotor** |  |  |  |  |  |  |
| pGGA004 | 35S (Cauliflower mosaic virus 35S) promoter | A-B | Ampicillin | 3550 bp | Lampropoulos et al. (2013) | Addgene ID 48814 |
|  |  |  |  |  |  |  |
| **Golden gate N-tags** |  |  |  |  |  |  |
| pGGB003 | B-dummy (default random sequence if no specific N-tag is desired) | B-C | Ampicillin | 2710 bp | Lampropoulos et al. (2013) | Addgene ID 48821 |
|  |  |  |  |  |  |  |
| **Golden gate C-tags** |  |  |  |  |  |  |
| pGGD001 | linker-GFP | D-E | Ampicillin | 3507 bp | Lampropoulos et al. (2013) | Addgene ID 48833 |
| pGGD002 | D-dummy (default random sequence with stop codon if no specific C-tag is desired) | D-E | Ampicillin | 2732 bp | Lampropoulos et al. (2013) | Addgene ID 48834 |
|  |  |  |  |  |  |  |
| **Golden gate plant terminator** |  |  |  |  |  |  |
| pGGE001 | RBCS terminator (from pea) | E-F | Ampicillin | 3333 bp | Lampropoulos et al. (2013) | Addgene ID 48839 |
|  |  |  |  |  |  |  |
| **Coding sequences** |  |  |  |  |  |  |
| pGG-C-Cas9PTA*-D | *Cas9-SV40* with stop codon | C-D | Ampicillin | 6828 bp | Houbaert et al. (2018) |  |
| pGG-C-Cas9PTA-D | *Cas9-SV40* | C-D | Ampicillin | 6825 bp | (Decaestecker et al., 2019) |  |
|  |  |  |  |  |  |  |
| **Unarmed gRNA entry vectors** |  |  |  |  | Decaestecker et al. (2019) |  |
| pGG-F-AtU6-26-BbsI-BbsI-G | AtU6-26 promoter and 'unarmed' gRNA scaffold | F-G | Ampicillin | 3177 bp | Decaestecker et al. (2019) |  |
| pGG-F-AtU6-26-AarI-AarI-G | AtU6-26 promoter and 'unarmed' gRNA scaffold | F-G | Ampicillin | 3183 bp | Decaestecker et al. 2019 |  |
|  |  |  |  |  |  |  |
| **Destination vector** |  |  |  |  |  |  |
| pEN-L1-AG-L2 |  | A-G | Kanamycin | 3990 bp | Houbaert et al. (2018) |  |
| pEN-L1-AG-L2,\(-Bbs1) |  | A-G | Kanamycin | 3990 bp | This work |  |
|  |  |  |  |  |  |  |
| **Unarmed gRNA destination vectors** |  |  |  |  |  |  |
| pCDB-Cas9 |  |  | Kanamycin | 8789 bp | This work |  |
| pCDB-Cas9-GFP |  |  | Kanamycin | 9567 bp | This work |  |
|  |  |  |  |  |  |  |
| **Unarmed gRNA destination vectors** (ccdB+) |  |  |  |  |  |  |
| pCDB-Cas9-ccdB |  |  | Kanamycin | 10206 bp | This work |  |
| pCDB-Cas9-GFP-ccdB |  |  | Kanamycin | 10978 bp | This work |  |
|  |  |  |  |  |  |  |
| **Expression vectors** |  |  |  |  |  |  |
| pCDB-Cas9-PDS |  |  | Kanamycin | 8791 bp | This work |  |
| pCDB-Cas9-GFP-PDS |  |  | Kanamycin | 9559 bp | This work |  |
| pCDB-Cas9-GAS |  |  | Kanamycin | 8791 bp | This work |  |
| pCDB-Cas9-GAO |  |  | Kanamycin | 8791 bp | This work |  |
| pCDB-Cas9-COS |  |  | Kanamycin | 8791 bp | This work |  |
|  |  |  |  |  |  |  |
| **Inserts** |  |  |  |  |  |  |
| ccdB insert | ccdB gene |  |  |  | Decaestecker et al. (2019) |  |
|  |  |  |  |  |  |  |
| **Other** |  |  |  |  |  |  |
| pKAR6 | GFP vector without NLS signal |  | Carbenicillin | 4450 bp | Robert Blanvillain, unpublished data |  |

Supplementary Table 3. Primer sequences used for HiPlex amplicon construction and ddPCR.

| **Primer** | **Sequence** | **Gene** | **Application** |
| --- | --- | --- | --- |
| Primer1 | 5‘-TTCAAGCACACAGATCACTTCA-3’ | PDS_F | HiPlex |
| Primer2 | 5’-TGGTGTAAAAGAACGGGCACT-3’ | PDS_R | HiPlex |
| Primer3 | 5’-GATGAGTTTGATCTATACACAACTTCT-3’ | GAS_F | HiPlex |
| Primer4 | 5’-ATGGTGTGTCATGGTTGATCA-3’ | GAS_R | HiPlex |
| Primer5 | 5’-ATGATCGCCACCATATTGAGCA-3’ | GAO_F | HiPlex |
| Primer6 | 5’-TTGAAGGAAAGATATCAGCTACATCG-3’ | GAO_R | HiPlex |
| Primer7 | 5’-GGCGAATACTGGAGGCAGAT-3’ | COS_F | HiPlex |
| Primer8 | 5’-AGTTGATGGGTGTTCCTGCT-3’ | COS_R | HiPlex |
| Primer 9 | 5’- TGCTTACCCTAGTGCCTCTGA-3’ | PP2AA3_F | ddPCR |
| Primer 10 | 5’- TTCCCAAATTTGTAGCAGCA-3’ | PP2AA3_R | ddPCR |
| Primer 11 | 5’- CCGCCATTAACTCAAGTGGA-3’ | PDS_F | ddPCR |
| Primer 12 | 5’- TTGGGAATTGCAATCTGTGA-3’ | PDS_R | ddPCR |

| **Supplementary Table 4**. Two HiPlex detected *CiPDS* loci in all screened regenerated (control and albino) plants. Purple = PAM site, green = gRNA target site, dashed vertical line = cut site, blue = SNP. |
| --- |
| \| ***CiPDS*** \| \| \| --- \| --- \| \| Locus 1 \| AGACTGTTTCAACATCACCTCAGGACAAAAAGATGTACTCTCATTTGGATGCTGTGATGCCATGGGTCACAGATTGCAATTC \| \| Locus 2 \| AGATTGTTTCAACATCACCTCAGGACAAAAAGATGTACTCTCATTTGGATGCTGTGATGCCATGGGTCACAGATTGCAATTC \| |

| **Supplementary Table 5**. Raw droplet digital PCR (ddPCR) data to quantify the copy number of *CiPDS* in a diploid ‘Van Hamme’ witloof plant. *, Deviating droplet profile; sample was excluded from the analysis. |
| --- |
| \| **#** \| **Sample ID** \| **Target** \| **Positive droplets** \| **Negative droplets** \| **Total droplets** \| **Concentration (copies.µL^-1^)** \| \| --- \| --- \| --- \| --- \| --- \| --- \| --- \| \| 1 \| NTC \| *CiPDS* \| 0 \| 17558 \| 17558 \| 0 \| \| 2 \| NTC \| *CiPDS* \| 0 \| 16678 \| 16678 \| 0 \| \| 3 \| Van Hamme 1 \| *CiPDS* \| 11696 \| 5140 \| 16836 \| 1396 \| \| 4 \| Van Hamme 1 \| *CiPDS* \| 12272 \| 5465 \| 17737 \| 1385 \| \| 5 \| Van Hamme 2 \| *CiPDS* \| 12016 \| 5812 \| 17828 \| 1319 \| \| 6 \| Van Hamme 2 \| *CiPDS* \| 11607 \| 5348 \| 16955 \| 1357 \| \| 7 \| Van Hamme 3 \| *CiPDS* \| 11409 \| 6053 \| 17462 \| 1246 \| \| 8 \| Van Hamme 3 \| *CiPDS* \| 11001 \| 5683 \| 16684 \| 1267 \| \| 9 \| Van Hamme 4 \| *CiPDS* \| 9721 \| 7490 \| 17211 \| 979 \| \| 10 \| Van Hamme 4 \| *CiPDS* \| 9584 \| 7303 \| 16887 \| 986 \| \| 11 \| NTC \| *PP2AA3* \| 0 \| 18601 \| 18601 \| 0 \| \| 12 \| NTC \| *PP2AA3* \| 0 \| 18648 \| 18648 \| 0 \| \| 13 \| Van Hamme 1 \| *PP2AA3* \| 4805 \| 15194 \| 19999 \| 323 \| \| 14 \| Van Hamme 1 \| *PP2AA3* \| 4013 \| 13268 \| 17281 \| 311 \| \| 15 \| Van Hamme 2 \| *PP2AA3* \| 4015 \| 13693 \| 17708 \| 303 \| \| 16 \| Van Hamme 2 \| *PP2AA3* \| 2313* \| 11972* \| 14285* \| 208* \| \| 17 \| Van Hamme 3 \| *PP2AA3* \| 4126 \| 14061 \| 18187 \| 303 \| \| 18 \| Van Hamme 3 \| *PP2AA3* \| 3776 \| 14027 \| 17803 \| 280 \| \| 19 \| Van Hamme 4 \| *PP2AA3* \| 2793 \| 14763 \| 17556 \| 204 \| \| 20 \| Van Hamme 4 \| *PP2AA3* \| 3063 \| 15287 \| 18350 \| 215 \| \| 21 \| NTC \| *UBQ10* \| 0 \| 17651 \| 17651 \| 0 \| \| 22 \| NTC \| *UBQ10* \| 1 \| 16842 \| 16843 \| 0.07 \| \| 23 \| Van Hamme 1 \| *UBQ10* \| 6812 \| 10860 \| 17672 \| 573 \| \| 24 \| Van Hamme 1 \| *UBQ10* \| 6680 \| 10226 \| 16906 \| 591 \| \| 25 \| Van Hamme 2 \| *UBQ10* \| 6841 \| 10549 \| 17390 \| 588 \| \| 26 \| Van Hamme 2 \| *UBQ10* \| 6342 \| 9603 \| 15945 \| 597 \| \| 27 \| Van Hamme 3 \| *UBQ10* \| 5768 \| 10663 \| 16431 \| 509 \| \| 28 \| Van Hamme 3 \| *UBQ10* \| 5954 \| 10103 \| 16057 \| 545 \| \| 29 \| Van Hamme 4 \| *UBQ10* \| 4820 \| 10753 \| 15573 \| 436 \| \| 30 \| Van Hamme 4 \| *UBQ10* \| 4851 \| 10693 \| 15544 \| 440 \| |
